# Shared Etiology of Psychotic Experiences and Depressive Symptoms in Adolescence: A Longitudinal Twin Study

**DOI:** 10.1093/schbul/sbw021

**Published:** 2016-03-18

**Authors:** Helena M. S. Zavos, Thalia C. Eley, Philip McGuire, Robert Plomin, Alastair G. Cardno, Daniel Freeman, Angelica Ronald

**Affiliations:** ^1^Department of Psychology, Institute of Psychiatry, Psychology and Neuroscience, King’s College London, London, UK;; ^2^Medical Research Council, Social, Genetic and Developmental Psychiatry Centre, Institute of Psychiatry, Psychology and Neuroscience, King’s College London, London, UK;; ^3^Institute of Psychiatry, Psychology, and Neuroscience, King’s College London, London, UK;; ^4^Academic Unit of Psychiatry and Behavioural Sciences, University of Leeds, Leeds, UK;; ^5^Department of Psychiatry, University of Oxford, Oxford, UK;; ^6^Centre for Brain and Cognitive Development, Birkbeck, Department of Psychological Sciences, University of London, London, UK

**Keywords:** psychotic experiences, depression, adolescence, twin study, genetics

## Abstract

Psychotic disorders and major depression, both typically adult-onset conditions, often co-occur. At younger ages psychotic experiences and depressive symptoms are often reported in the community. We used a genetically sensitive longitudinal design to investigate the relationship between psychotic experiences and depressive symptoms in adolescence. A representative community sample of twins from England and Wales was employed. Self-rated depressive symptoms, paranoia, hallucinations, cognitive disorganization, grandiosity, anhedonia, and parent-rated negative symptoms were collected when the twins were age 16 (*N* = 9618) and again on a representative subsample 9 months later (*N* = 2873). Direction and aetiology of associations were assessed using genetically informative cross-lagged models. Depressive symptoms were moderately correlated with paranoia, hallucinations, and cognitive disorganization. Lower correlations were observed between depression and anhedonia, and depression and parent-rated negative symptoms. Nonsignificant correlations were observed between depression and grandiosity. Largely the same genetic effects influenced depression and paranoia, depression and hallucinations, and depression and cognitive disorganization. Modest overlap in environmental influences also played a role in the associations. Significant bi-directional longitudinal associations were observed between depression and paranoia. Hallucinations and cognitive disorganization during adolescence were found to impact later depression, even after controlling for earlier levels of depression. Our study shows that psychotic experiences and depression, as traits in the community, have a high genetic overlap in mid-adolescence. Future research should test the prediction stemming from our longitudinal results, namely that reducing or ameliorating positive and cognitive psychotic experiences in adolescence would decrease later depressive symptoms.

## Introduction

Identifying those at high risk of psychosis is an important part of early clinical intervention in mental health.^1^ One area of study has focused on psychotic experiences which are present in the general population in adolescence.^2,3^ Psychotic experiences in adolescence have been shown to be associated with elevated rates of prodromal syndromes^2^ and increased risk of psychosis in later life^4^ although another study did not find an association with psychotic disorders at age 18 years.^5^ The presentation of psychosis is heterogeneous, including positive (eg, hallucinations) and negative (eg, lack of affect) symptoms. Both patients with psychotic disorders and people at high risk of psychosis often experience depressive, as well as psychotic symptoms.^1^

The precise role of depression in psychosis is unclear. It has been posited as a vulnerability factor, a predictor of transition, a maintenance factor, and as a response to experiencing a psychotic disorder.^6^ A number of population based cross-sectional studies have found significant associations between low mood and psychotic-like experiences.^7–10^ A high prevalence of depression and anxiety disorders is also evident in individuals at high risk for psychosis.^11^ Depressive disorders have also been associated with an increased risk of later transition to psychosis in high-risk individuals.^12^ The role of depression in the maintenance of paranoia is supported by prospective studies of individuals with psychosis. For example, in one study in adults, depressed mood predicted paranoid symptoms when controlling for baseline symptoms of paranoia with no evidence for the opposite direction of effects (paranoid symptoms did not predict depressed mood).^13^ Therefore there is support for the idea of depression contributing towards maintenance and prediction of relapse in psychotic disorders. However, the majority of these studies were conducted on individuals with a psychotic disorder or who had presented to clinical services in a high risk state. It is therefore unclear how depression and psychotic experiences are related earlier in development prior to the onset of psychotic disorders. Compared to general population samples, clinical samples are known to have inflated levels of comorbidity, which will introduce bias in the results. Furthermore, effects of treatment are difficult to control for in studies of comorbid symptoms in clinical samples. In the only study to explore bidirectional effects between psychotic experiences and depression in adolescence, psychotic experiences at 12 years old predicted depression at 18 years old to a greater extent than depression at 12 years old predicted psychotic experiences at 18 years old.^14^

Studies looking at the aetiology of psychotic experiences and depression separately have shown that both genetic and environmental influences are important. Heritability in adolescents has been estimated between 15%–59% for psychotic experiences^15–17^ and 40%–45% for depression symptoms.^18–20^ Although a distinction is often made between psychosis and “neurotic” disorders, molecular genetic and family studies have suggested a considerable degree of overlap in the genetic causes of mood and psychotic disorders.^21–23^ A recent study using genome-wide genotype data from the Psychiatric Genomics Consortium (PGC) estimated the genetic correlation using common SNPs to be .43 between schizophrenia and major depression.^24^ The degree of genetic and environmental overlap between the full range (including positive, negative, and cognitive symptoms) of psychotic experiences and depressive symptoms earlier in development is unknown.

**Fig. 1.**
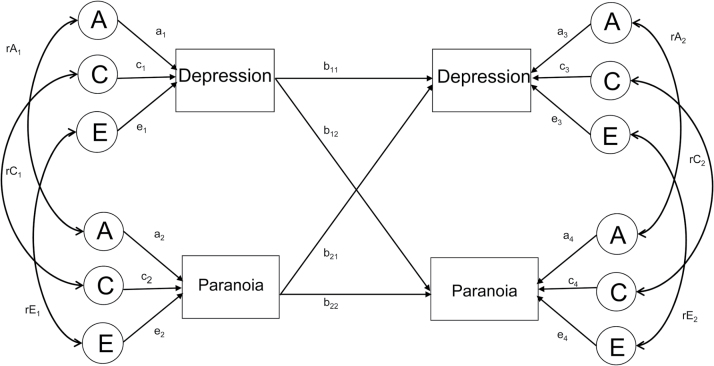
Example path diagram of the cross-lagged model. A = additive genetic effects; C = shared environmental effects; E = non-shared environmental effects. Variance paths, which must be squared to estimate the proportion of variance accounted for, are represented by lowercase letters and followed by 1 subscripted numeral eg, a_1_, c_1_, e_1_. Genetic, shared, and non-shared environmental correlations are represented by rA, rC, and rE, respectively and are followed by 1 lowercase numeral. Phenotypic partial coefficients are represented by lowercase letters followed by subscripted numerals eg, b_11_, b_12_ (1, time point 1; 2, time point 2).

The first aim of the current study was to investigate longitudinal associations between the full range of psychotic experiences and depressive symptoms in an adolescent community twin sample. A symptom-specific approach to studying the development and course of psychotic experiences was taken as psychotic symptoms do not correlate strongly and fall into multiple principal components.^3,25^ The use of a community sample of adolescents allowed us to examine the full range of the psychotic and depression traits and to understand the manifestations of these problems prior to the onset of depressive and psychotic disorders. Our second aim was to assess the degree to which psychotic experiences and depressive symptoms co-vary in the general population due to common genetic and environmental influences. The study aims were addressed using a genetically sensitive cross-lagged model (figure 1). These models estimate the phenotypic longitudinal effects of all variables on each other, while controlling for the concurrent (genetic and environmental) associations between them. The direction of effect between variables can be established by evaluating the strength and confidence intervals of the longitudinal effects.

## Method

### Participants

The Longitudinal Experiences And Perceptions (LEAP) study assessed psychotic experiences in adolescents^3^ drawn from the Twins Early Development Study (TEDS), a general population sample of monozygotic (MZ) and dizygotic (DZ) twins born in England and Wales between 1994–1996.^3^ TEDS was approved by the Institute of Psychiatry ethics committee. TEDS had originally contacted a sample of 16 302 sets of parents after they had twins born in 1994–1996, of whom 13 488 families responded with a written consent form. Families were not contacted for the LEAP study if they had withdrawn from TEDS, had never returned any data, had known address problems, or were special cases, most notably medical exclusions (eg, cerebral palsy; any genetic, chromosomal or inherited disorder; brain damage or disorders affecting brain function; Downs syndrome; profound deafness; global developmental delay; complete blindness; death of either twin). Zygosity was assigned using a parent-reported questionnaire of physical similarity at ages 1, 3 and 4, which is over 95% accurate when compared to DNA testing.^26^ For cases where zygosity was unclear or where there was DNA data available, DNA testing was conducted.^27^

#### Time 1 Assessment.

Initially, 10 874 TEDS families were contacted and invited to participate in LEAP time 1. Of those contacted, 5076 (47%) parents provided data and 5059 (47%) twin pairs provided data (*M* = 16.32 y; SD = 0.68 y). Individuals were excluded from the analyses (*N* = 610), if they had severe medical disorder, if they had experienced severe perinatal complications (low birth weight; short gestational age; maternal drinking during pregnancy; long period of special care after birth; long stay in hospital after birth) or if their zygosity was unknown. The twin sample after exclusions (*N* = 4743 families) was 45% male. Demographic information on the participating and nonparticipating families is given in the supplementary material.

#### Time 2 Assessment.

At time 2, a representative subsample of 1773 of the participating families were contacted and invited to participate again on average 9 months later. Data from 1464 families (83%) were obtained from both parents and twins (*M* = 17.06 y; SD = 0.88 y).

### Measures

#### Specific Psychotic Experiences Questionnaire.

The Specific Psychotic Experiences Questionnaire (SPEQ)^3^ assesses 6 types of psychotic experiences in adolescents: Paranoia (15 items, example item: “I can detect coded messages about me in the press/TV/internet”), Hallucinations (9 items, example item: “Hear noises or sounds when there is nothing about to explain them”), Cognitive Disorganization (11 items, example item: “Do you often have difficulties in controlling your thoughts?”), Grandiosity (8 items, example item: “Everyone is going to know about me because of my greatness”), Hedonia (10 items which was reverse coded to indicate level of anhedonia, example item: “I look forward to a lot of things in my life”), all via self report, and Negative Symptoms via parent report (10 items, example item: “Seems emotionally “flat,” rarely changes the emotions s/he shows”). The SPEQ was developed by selecting and combining items from existing scales for adults and adapting to be age appropriate. Age appropriateness of items was ensured via obtaining expert clinical opinion (D.F., A.G.C., and P.M.) and via piloting on this age group (described in Ronald et al^3^). Subscales show good to excellent internal consistency (Cronbach’s α = .77–.93), and the positive and cognitive subscales have been validated against the Psychosis-Like Symptoms (PLIKS) measure.^3,5^ For all the SPEQ subscales except Anhedonia and Hallucinations, individuals who reported a family history (having a first- or second-degree relative with schizophrenia or bipolar disorder) scored higher than individuals without a family history of psychosis (all *P* < .05 except Hallucinations which showed a trend in this direction). Full details about the measures of psychotic experiences are described elsewhere.^3^

#### Depression Symptoms.

Depressive symptoms were measured using the Short Mood and Feelings Questionnaire,^28^ a 13-item self-report measure assessing how often depressive symptoms occurred in the past 2 weeks (example item: “I felt miserable or unhappy”). Responses were summed to give total depressive symptoms scores, in line with research suggesting a single underlying continuum of severity of depressive symptoms using this measure.^29^ The measure demonstrates good reliability and validity (time 1 α = .88; time 2 α = .92).^28^ It can also discriminate between the individuals with depression and healthy controls^30,31^ and is suitable for use in adolescents.^32^

#### The Twin Design.

The twin design aims to segregate phenotypic variance into 3 components: additive genetic influences (A), shared environment (C), and non-shared environment (E).^33^ Additive genetic influences (A) refer to effects of alleles or loci which act in an additive, rather than dominant, manner (2 copies of a risk allele at the same locus confer twice the risk of 1 copy). Shared environment (C) refers to aspects of the environment which make members of the same family similar to one another, and non-shared environment (E) refers to environmental effects which make members of the same family different from one another. The twin method compares the degree of resemblance between pairs of MZ twins who share all of their DNA and all their shared environment (if they grow up in the same family) and DZ twins who share on average 50% of their DNA and all of their shared environment. Genetic influences on a trait are inferred if MZ correlations are greater than DZ correlations as this increased similarity in MZ twins can only be accounted for by their increased genetic resemblance. Within-pair similarity that is not due to genetic factors is attributed to shared environmental influences (C) and would be implicated if the DZ correlation is greater than half that of the MZ correlation for a given trait. Non-shared environment (E) accounts for individual specific environmental factors that create differences among siblings from the same family. These are estimated from within-pair differences between MZ twins as E is the only influence that makes MZ twins different from one another. Measurement error is also included in this E term. Missing data was handled using full information maximum.^34^ Genetic analyses were conducted using structural equation modeling program Mx.^35^

#### Cross-lagged Model.

Genetically sensitive cross-lagged models estimate longitudinal associations between variables (in our case between times 1 and 2) while controlling for the concurrent (genetic and environmental) associations between all variables in the model at time 1. Genetic cross-lagged models were run when phenotypic correlations between specific psychotic experiences and depression were above .3 and significant. Overall, this type of model can be used to extract the following types of information (figure 1):

#### Within-time Genetic and Environmental Influences.

At both time 1 and 2, univariate estimates of the magnitude of genetic and environmental influences on psychotic experiences and depression can be calculated. For example, time 1 depression is influenced by genes (a_1,_), shared environment (c_1_), and non-shared environment (e_1_). Genetic and environmental influences on time 2 variables can be calculated in a similar manner.

#### Within-time Genetic and Environmental Covar iation.

Genetic and environmental influences on the associations between depression and psychotic experiences can also be estimated. This is done separately at times 1 and 2. For example, the genetic (rA_1_), shared environment (rC_1_), and non-shared environment (rE_1_) between depression and paranoia are estimated at time 1. In addition to the genetic and environmental correlations, it is also possible to calculate the proportion of the phenotypic correlation due to genes or environment.

#### Cross-time Phenotypic Associations.

The cross-time associations are represented as partial regression coefficients. The value of each path is independent of the other associations addressed in the model. The prediction of a trait by the same trait (b_11_, b_22_), as well as the prediction of a trait by another trait (b_12_, b_21_), can be assessed. The value of b_11_, eg, is the contribution of time 1 depression to time 2 depression. The value of b_12_ indexes the contribution of time 1 depression to time 2 paranoia independent of the within-time relationship between depression and paranoia at time 1.

## Results

### Descriptive Statistics

Descriptive statistics for all study variables at times 1 and 2 are given in table 1 and have been reported previously.^3,15^ At time 1, females scored significantly higher than males on paranoia, hallucinations, cognitive disorganization and depression; males scored significantly higher than females on grandiosity, anhedonia, and parent-rated negative symptoms. The only difference at time 2 was that females rather than males scored significantly higher for anhedonia and there were no significant sex differences in hallucinations.

**Table 1.** Descriptive Statistics for Depression and Psychotic Experiences at Times 1 and 2

	*N*	Range	α	Total	Males	Females	*t* (*df*)	*P*-value
				Mean (SD)	Mean (SD)	Mean (SD)		
Time 1								
Paranoia	4798	0–72	.93	12.17 (10.62)	11.76 (10.43)	12.50 (10.77)	2.37 (4796)	.02
Hallucinations	4806	0–45	.87	4.66 (6.01)	4.30 (5.77)	4.95 (6.19)	3.77 (4804)	<.00
Cognitive disorganization	4799	0–11	.77	3.96 (2.85)	3.40 (2.72)	4.41 (2.87)	12.33 (4797)	<.00
Grandiosity	4802	0–24	.85	5.32 (4.43)	5.82 (4.57)	4.40 (4.25)	−7.14 (4800)	<.00
Anhedonia	4802	0–49	.78	16.33 (7.93)	18.49 (8.00)	14.59 (7.44)	−17.47 (4800)	<.00
Negative symptoms	4817	0–30	.85	2.81 (3.89)	3.17 (4.09)	2.51 (3.67)	−5.91 (4815)	<.00
Depression	4806	0.26	.88	3.60 (4.42)	2.64 (3.49)	4.37 (4.91)	13.75 (4804)	<.00
Time 2								
Paranoia	1437	0–72	.95	14.82 (13.88)	13.72 (13.21)	15.64 (14.30)	2.60 (1435)	.01
Hallucinations	1439	0–45	.90	6.79 (7.60)	6.67 (7.76)	6.89 (7.48)	0.54 (1437)	.59
Cognitive disorganization	1440	0–11	.80	4.49 (3.11)	3.94 (2.98)	4.90 (3.13)	5.83 (1438)	<.00
Grandiosity	1439	0–24	.89	4.76 (4.78)	5.60 (5.10)	4.14 (4.44)	−5.82 (1437)	<.00
Anhedonia	1441	0–47	.79	17.03 (8.08)	15.42 (7.59)	19.23 (8.21)	−2.19 (1428)	.03
Negative symptoms	1437	0–30	.89	3.72 (4.75)	4.24 (5.02)	15.64 (14.30)	−3.50 (1430)	<.00
Depression	1439	0.26	.92	4.37 (5.62)	3.10 (4.28)	5.31 (6.27)	7.53 (1437)	<.00

*Note*: *N*, number of individuals; *t*, *t* statistic; *df*, degrees of freedom. Mean and SD for 1 randomly selected member of a twin pair.

Within-time and cross-time correlations between specific psychotic experiences and depression are presented in table 2. Moderate correlations, within and cross-time, were evident between depression and paranoia, depression and hallucinations, and depression and cognitive disorganization (*r* = .34–.60; *P* < .001). Lower correlations were observed between depression and anhedonia, and depression and parent rated negative symptoms (*r* = .12–.24; *P* < .001). Nonsignificant correlations, within and cross time, were observed for depression and grandiosity (*r* = −.04–.00; *P* > .05). Overall 3 relationships were of adequate strength to consider for twin modeling (paranoia and depression; hallucinations and depression; cognitive disorganization; and depression).

**Table 2.** Phenotypic Correlations Between Psychotic Experiences and Depression Symptoms

	Time 1 Depression		Time 2 Depression	
	*N*	*r* (95% CI)	*N*	*r* (95% CI)
Time 1				
Paranoia	4796	.53 (0.51–0.55)*	1437	.40 (0.36–0.44)*
Hallucinations	4805	.41 (0.38–0.43)*	1439	.37 (0.32–0.41)*
Cognitive disorganization	4796	.54 (0.52–0.56)*	1436	.47 (0.42–0.51)*
Grandiosity	4800	−.01 (−0.04–0.02)	1437	.00 (−0.05–0.05)
Anhedonia	4800	.13 (0.10–0.16)*	1438	.12 (0.07–0.17)*
Negative symptoms (P)	4771	.19 (0.16–0.22)*	1437	.13 (0.08–0.18)*
Time 2				
Paranoia	1436	.49 (0.45–0.53)*	1436	.60 (0.56–0.63)*
Hallucinations	1438	.34 (0.30–0.39)*	1438	.39 (0.35–0.43)*
Cognitive disorganization	1439	.51 (0.47–0.54)*	1438	.57 (0.53–0.60)*
Grandiosity	1438	−.01 (−0.06–0.04)	1437	−.04 (−0.10–0.01)
Anhedonia	1440	.12 (0.07–0.17)*	1439	.17 (0.12–0.22)*
Negative symptoms (P)	1431	.21 (0.16–0.26)*	1424	.24 (0.19–0.29)*

*Note*: *N*, number of observations; P, parent report. Within-time correlations on diagonal; cross time correlations on off diagonal. Correlations conducted on 1 randomly selected member of a twin pair.

*P < .001.

### Cross-lagged Model

First, as is standard twin model-fitting procedure,^33^ a saturated model which allowed variances, covariances, and means to vary by zygosity group, as well as sex was run, to get a baseline index of fit. Models which allowed for sex differences in A, C, and E parameters, and more conservative models which did not, were run. Models which allowed for sex differences had a better fit than those that did not (see supplementary table 1 for fit statistics); however, as paths were similar across the sexes and Bayesian Information Criterion (BIC) was lower in no sex difference models, for simplicity we present results from the no sex difference model in figure 1 (full sex difference models are shown supplementary figure 1).

### Within-time Genetic and Environmental Influences

**Fig. 2.**
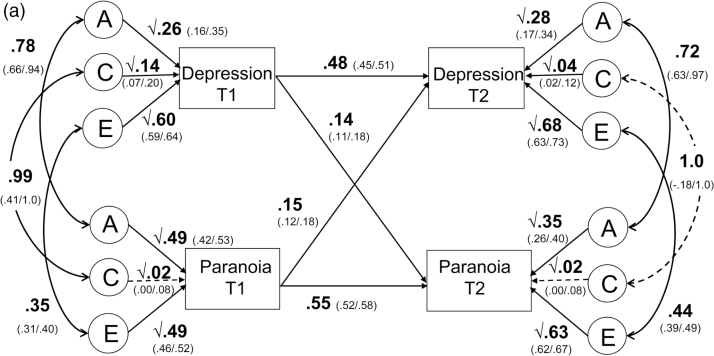

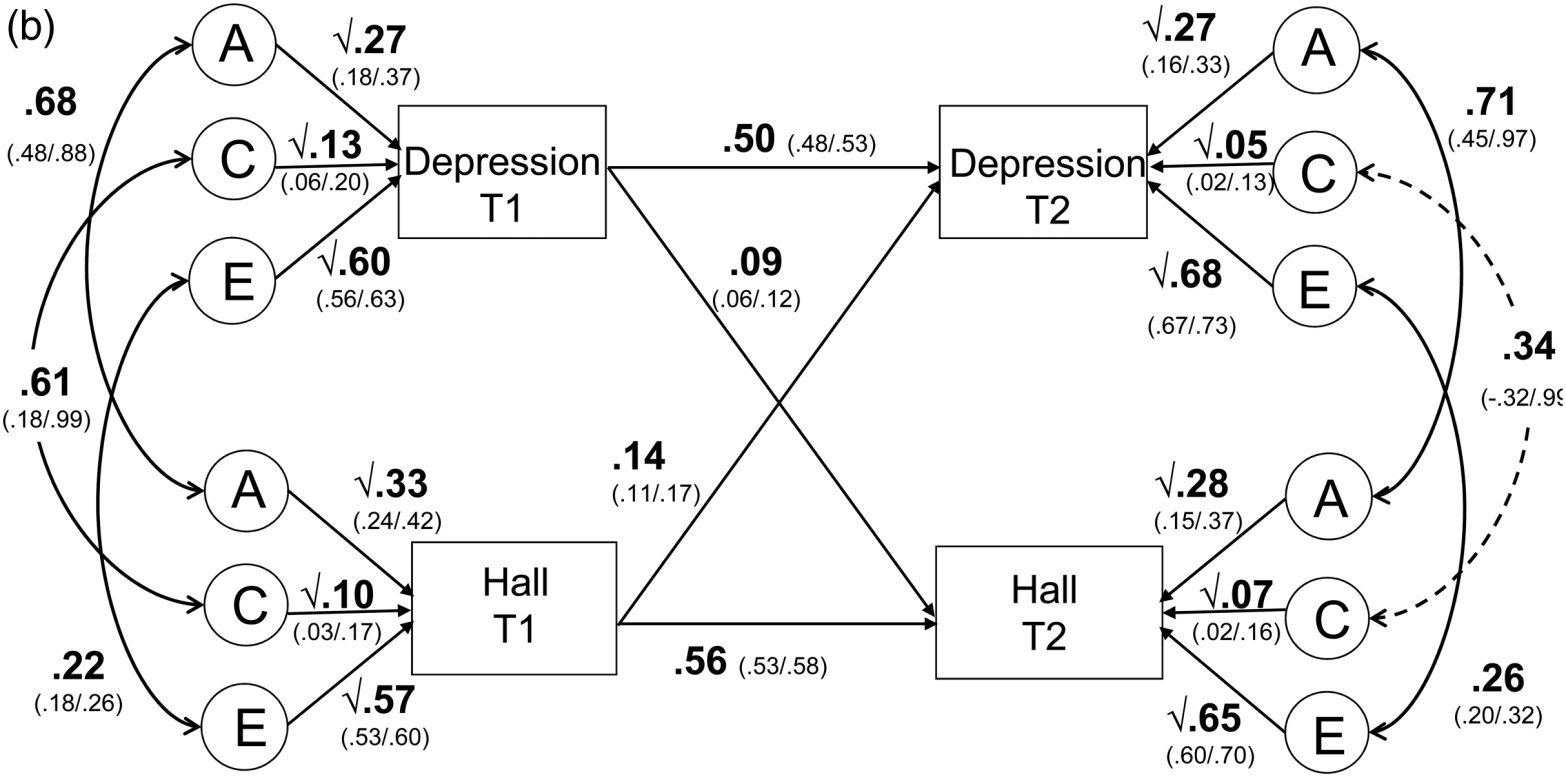

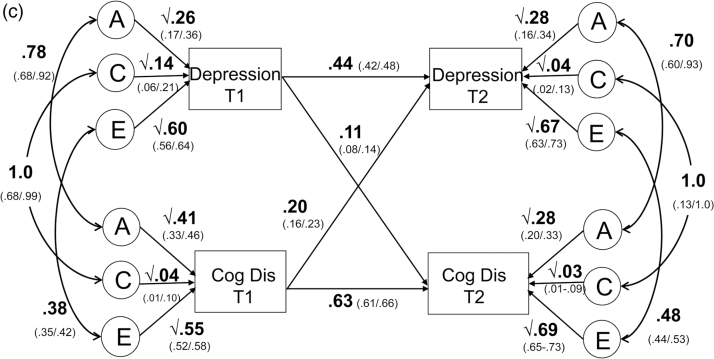
Bivariate cross-lagged models for depression and psychotic experiences. (a) Longitudinal relationship between depression symptoms and paranoia. (b) Longitudinal relationship between depression symptoms and hallucinations. (c) Longitudinal relationship between depression symptoms and cognitive disorganization. *Note*: Hall: hallucinations; cog dis: cognitive disorganization. Additive genetic (A), shared (C), and non-shared environmental (E) influences on total variance at time 2 presented. Correlations between latent factors at time 2 are presented on total, rather than residual, variances.

Cross-twin correlations and univariate genetic and environmental estimates are presented in supplementary table 2. All variables were moderately heritable, with low shared environmental and modest non-shared environmental influences, as reported elsewhere.^15,36^ Genetic and environmental influences at both times, along with cross-sectional genetic and environmental correlations, are shown in figure 2. For example, 26% of the variance in depression at time 1 was due to genetic influences, 14% due to shared environmental influences and 60% due to non-shared environmental influences.

### Within-time Genetic and Environmental Covariation

Genetic correlations (rA) at time 1 were high between paranoia and depression (rA = .78), hallucinations and depression (rA = .68) and cognitive disorganization and depression (rA = .78). Shared environmental correlations were generally high between the specific psychotic experiences and depression; however, as C explained very little of variance in measures this should not be over-interpreted. Non-shared environmental correlations were moderate (rE = .22–.38). Patterns of genetic and environmental correlations were similar at time 2. Genetic influences accounted for between 35%–53% and non-shared environmental influences accounted for between 32%–59% of the phenotypic correlation (table 3). Shared environment accounted for little of the covariation (table 3).

**Table 3.** Bivariate Heritabilities and Environmental Estimates Between Depression and Psychotic Experiences

	Depression			
	*r*Ph	Bivariate a^2^	Bivariate c^2^	Bivariate e^2^
Time 1				
Paranoia	.53 (0.51–0.54)	.53 (0.40–0.65)	.11 (0.01–0.21)	.36 (0.32–0.41)
Hallucination	.40 (0.38–0.42)	.51 (0.33–0.68)	.17 (0.04–0.31)	.32 (0.25–0.38)
Cognitive disorganization	.56 (0.54–0.57)	.46 (0.33–0.58)	.14 (0.05–0.24)	.40 (0.35–0.45)
Time 2				
Paranoia	.54 (0.52–0.57)	.42 (0.30–0.52)	.05 (−0.01–0.13)	.53 (0.47–0.59)
Hallucination	.39 (0.36–0.42)	.50 (0.29–0.67)	.05 (−0.06–0.20)	.45 (0.45–0.54)
Cognitive disorganization	.56 (0.54–0.59)	.35 (0.22–0.44)	.07 (0.01–0.15)	.59 (0.53–0.65)

*Note*. *r*Ph, phenotypic correlation. Bivariate genetic (a^2^), common environment (c^2^), and unique environment (e^2^) estimates the proportion of the phenotypic correlation between depression symptoms and psychotic experiences due to genetic and environmental influences. 95% CIs in parentheses.

### Cross-time Phenotypic Associations

#### Cross-time Within-Variable.

The within-variable continuity paths accounted for the largest proportion of variance in variables at time 2. Specifically, within-variable continuity from time 1 to time 2 was estimated at .48 for depression which suggests that 23% (.48^2^) of the variance of time 2 depression is explained by time 1 depression. Within-variable stability was also high for psychotic experiences (paranoia: .55, hallucinations: .56, cognitive disorganization: .63).

#### Cross-time Cross-Variable.

*Paranoia and Depression* (figure 2a) Cross-time cross-variable paths from paranoia to depression (.15) and from depression to paranoia (.14) were both significant and of a similar magnitude (confidence intervals overlapped), suggesting a reciprocal relationship between them over time.

*Hallucinations and Depression* (figure 2b) Cross-time cross-variable paths from hallucinations to depression (.14) and from depression to hallucinations (.09) were both significant. Of note, the path from hallucinations to depression was stronger than the converse association although confidence intervals overlapped. We tested whether these paths could be equated but found that this led to a significant deterioration in fit (χ^2^(1) = 29.63, *P* ≤ .01). This suggests hallucinations predict depression (after controlling for earlier depression) to a greater extent than depression predicts hallucinations (after controlling for earlier hallucinations).

*Cognitive Disorganization and Depression* (figure 2c) Time 1 cognitive disorganization significantly predicted time 2 depression (.20). The converse association (time 1 depression to time 2 cognitive disorganization) whilst significant was half the magnitude (.11). Confidence intervals did not overlap, suggesting this difference in magnitude was significant. This was further examined using a χ^2^ difference test by equating the paths of interest. Equating the 2 paths led to a significant deterioration in fit (χ^2^(1) = 53.68, *P* ≤ .01). This suggests that cognitive disorganization impacts depression across time, after controlling for earlier levels of depression, and is a significantly stronger effect than that of depression on later cognitive disorganization (after controlling for earlier levels of cognitive disorganization).

## Discussion

Depressive symptoms were moderately correlated with paranoia, hallucinations, and cognitive disorganization in adolescence. Largely the same genetic influences make individuals vulnerable to traits of depression and positive and cognitive psychotic experiences in adolescence, suggesting that the pleiotropic genetic effects reported in adult clinical samples^21–24^ between major depression and psychotic disorders are also true of psychotic experiences and depression symptoms in the community in adolescence.

Strong correlations were found between positive psychotic experiences and depressive symptoms in line with previous literature.^7–10^ Correlations between negative psychotic experiences and depression symptoms were lower although they were still significant. The low correlation between negative symptoms and depression may be due to the fact that negative symptoms reflect a lack of affect as opposed to depression, which involves heightened/changed affect. Different raters were used to assess negative symptoms (parent-rated) and depression symptoms (self-rated) in line with recommendations in the field regarding the optimal rater for these domains (observer ratings for negative symptoms, self-ratings for depression). However the use of different raters for these scales will have led to reduced observed correlation between them. Our measure of anhedonia focused on the anticipatory aspect of anhedonia (inability to experience pleasure for future events) rather than its consummatory component (inability to experience pleasure in the moment). Anticipatory anhedonia has been shown to be more important in schizophrenia as opposed to depression.^37–39^ Our measure of depression had only 1 item specifically addressing anhedonia (“I didn’t enjoy anything at all”). This may explain the low correlation between anhedonia and depression.

We found that symptoms of depression and positive and cognitive psychotic experiences impact each other over time, even after taking into account the shared genetic propensity that exists between them and existing associations at earlier ages. For both hallucinations and cognitive disorganization, the strength of the path from psychotic experiences to depression was stronger than the opposite direction. This is in contrast to findings in adult clinical samples which suggest the direction of effects is from depression to psychosis but not vice-versa.^13^ Interestingly, a recent study showed that psychotic experiences in mid-adolescence predict persistence of suicidal ideation in later adolescence.^40^ Our results are broadly in line with a longitudinal study of adolescents which found evidence that psychotic experiences predicted depression to a greater extent than vice versa.^14^ Our results however provide greater support for the role of depression in predicting future psychotic experiences than previous research in adolescence.^14^ These differences could be due to the distinct developmental time frames in the current study and the previous study. Future research should test the prediction stemming from our longitudinal results, namely that reducing or ameliorating positive and cognitive psychotic experiences in adolescence would decrease later depressive symptoms.

The high genetic correlations observed between psychotic experiences and depression suggests that 1 reason that psychotic experiences and depression co-occur in adolescence is due to shared aetiological influences which make individuals vulnerable to both sets of traits. The results suggest that the genetic relationship between psychotic experiences and symptoms of depression is established prior to adulthood. Although the same genetic and familial influences appear to operate across the continuum for psychotic experiences,^15,41^ the 2 studies thus far have not found that a schizophrenia polygenic risk score predicts having more psychotic experiences in adolescence.^42,43^

Non-shared environmental influences were important for psychotic experiences and depression symptoms in line with previous research in adolescence. Our results also show moderate non-shared environmental correlations between depression and psychotic experiences. This suggests that some of the same environmental factors that influence depression may also influence psychotic experiences. Such environments could include childhood adversity^44–46^ and stressful life events,^47,48^ which have been shown to be risk factors for both depressive and psychotic disorders.

The twin design is based on several assumptions and ideally findings should be replicated across multiple study designs (see Plomin et al^49^ for detail). Measures of psychotic experiences and depression were collected using self-report questionnaires: although the measures are well validated and show good internal consistencies, it is possible that they fail to capture the full complexities of the phenotypes. It is also possible that some individuals may interpret questions in alternative ways from that intended. Self-report data of psychotic experiences have also been shown to give higher means than interview data.^50^ The use of the same rater for psychotic experiences and depression will give higher correlations than using different raters across 2 phenotypes. The use of self-report measures allowed us to investigate the full range of positive (eg, grandiosity), negative (eg, lack of affect) and cognitive psychotic experiences (eg, disordered thinking) in a large sample with a narrow age range.^51^ Whilst all measures had excellent construct validity and were checked to ensure that items did not explicitly overlap, it is possible that some of the observed pleiotropic genetic effects could be due to items in the depression and psychotic experiences scales tapping into similar underlying constructs. As the current study was conducted in a community sample, our results were not confounded with effects of treatment and are not biased towards the most severe and comorbid cases. Limitations associated with the cross-lagged model need to be taken into account including stationarity, the assumption that causal processes influencing variables do not change over the time period in question (see Kenny^52^). All models fitted less well than the saturated model, however, poor fits are commonly found within all cross-lagged models in large samples.^53–55^ Future research could extend our work by exploring the relationship between psychotic experiences and depression across longer periods of time.

## Conclusion

Psychotic experiences, in particular positive and cognitive types, are strongly related to depressive symptoms in adolescence. Our study shows that the co-occurrence of depression and psychotic experiences is due to high genetic overlap and modest overlap in environmental influences, yet in addition, psychotic experiences and depression directly impact each other over time. While adolescent depression and paranoia predict each other bidirectionally over time, hallucinations and cognitive disorganization appear to impact later depression more so than vice-versa. The results of this study illustrate the way in which symptoms of depression and psychotic experiences in adolescence, both known risk factors for later psychiatric disorders, may develop and persist.

## Supplementary Material

Supplementary material is available at http://schizophreniabulletin.oxfordjournals.org.

## Funding

Medical Research Council (G1100559 to A.R., G0901245, G0500079 to R.P.). D.F. is supported by a UK Medical Research Council Senior Clinical Fellowship (G0902308). This article represents independent research part funded by the National Institute for Health Research (NIHR) Biomedical Research Centre at South London and Maudsley NHS Foundation Trust, and King’s College London. The views expressed are those of the author(s) and not necessarily those of the NHS, the NIHR or the Department of Health.

## Supplementary Material

Supplementary Data
