# Supplementary material for: Shared Etiology of Psychotic Experiences and Depressive Symptoms in Adolescence: A Longitudinal Twin Study
Source: Schizophr Bull. 2016 Mar 18;42(5):1197–206. doi: 10.1093/schbul/sbw021 (PMC4988737; doi:10.1093/schbul/sbw021)
Supplement: Supplementary Data [file supp_42_5_1197__index.html]

Shared Etiology of Psychotic Experiences and Depressive Symptoms in Adolescence: A Longitudinal Twin Study — Shared Etiology of Psychotic Experiences and Depressive Symptoms in Adolescence: A Longitudinal Twin Study — Supplementary Data 

# Shared Etiology of Psychotic Experiences and Depressive Symptoms in Adolescence: A Longitudinal Twin Study

## Supplementary Data

Data files

- Supplementary Data - Supplementary Data
