# Supplementary material for: Shared Etiology of Psychotic Experiences and Depressive Symptoms in Adolescence: A Longitudinal Twin Study
Source: Schizophr Bull. 2016 Mar 18;42(5):1197–206. doi: 10.1093/schbul/sbw021 (PMC4988737; doi:10.1093/schbul/sbw021)
Supplement: Supplementary Data [file supp_sbw021_Supplementary_materials.doc]

**Appendices**

**eTable 1.** Model fit for cross-lagged models

|  | Model | -2ll | df | Δχ^2^ | Δdf | p | AIC | BIC |
| --- | --- | --- | --- | --- | --- | --- | --- | --- |
| Paranoia-depression |  |  |  |  |  |  |  |  |
|  | Saturated | 66291.36 | 24723 |  |  |  |  |  |
|  | Cross-lagged sex differences | 66538.15 | 24897 | 246.799 | 174 | <0.01 | 16744.2 | -72347.19 |
|  | Cross-lagged no sex differences | 66600.7 | 24909 | 309.343 | 186 | <0.01 | 16782.7 | -72366.83 |
| Hallucinations-depression |  |  |  |  |  |  |  |  |
|  | Saturated | 58475.68 | 24736 |  |  |  |  |  |
|  | Cross-lagged sex differences | 58793.81 | 24910 | 318.132 | 174 | <0.01 | 8973.813 | -76274.51 |
|  | Cross-lagged no sex differences | 58856.8 | 24922 | 381.115 | 186 | <0.01 | 9012.796 | -76293.93 |
| Cognitive disorganisation-depression |  |  |  |  |  |  |  |  |
|  | Saturated | 46128.32 | 24725 |  |  |  |  |  |
|  | Cross-lagged sex differences | 46427.62 | 24899 | 299.299 | 174 | <0.01 | -3370.38 | -82419.05 |
|  | Cross-lagged no sex differences | 46468.03 | 24911 | 339.71 | 186 | <0.01 | -3353.97 | -82441.64 |

Note: No sex differences model allowed for variance differences across sex. -2LL = negative 2 log likelihood; df = degrees of freedom; Δ*X^2^*= likelihood ratio *X^2^* test comparing the -2LL fit of each model to the -2LL fit of the saturated model; Δdf = difference in degrees of freedom comparing each model to the saturated model; p = *p*-value; AIC = Akaike’s Information Criterion; BIC = Bayesian Information Criterion.

**eTable 2:** Cross-twin within-trait correlations and univariate genetic and environmental estimates for psychotic experiences and depression symptoms

|  | Cross-twin within trait correlations | | | | | Univariate Genetic and Environmental estimates | | |
| --- | --- | --- | --- | --- | --- | --- | --- | --- |
|  | MZM | DZM | MZF | DZF | DZOS | A | C | E |
| Time 1 | N=1426 | N=1334 | N=2047 | N=1788 | N=3023 |  |  |  |
| Paranoia | .47 (.41-.52) | .28 (.20-.34) | .55 (.51-.59) | .30 (.23-.35) | .24 (.19-.28) | .50 (.41-.54) | .01 (.00-.09) | .49 (.46-.52) |
| Hallucinations | .37 (.30-.43) | .27 (.20-.34) | .48 (.43-.53) | .33 (.27-.39) | .23 (.18-.28) | .32 (.22-.42) | .11 (.04-.19) | .57 (.53-.61) |
| Cognitive Disorganisation | .40 (.34-.46) | .30 (.23-.37) | .50 (.45-.54) | .20 (.13-.26) | .24 (.19-.28) | .43 (.33-.49) | .02 (.00-.10) | .55 (.51-.58) |
| Negative symptoms | .83 (.80-.85) | .53 (.47-.58) | .83 (.81-.85) | .59 (.55-.63) | .50 (.46-.54) | .59 (.54-.64) | .24 (.19-.29) | .17 (.16-.18) |
| Depression | .37 (.30-.43) | .22 (.14-.29) | .42 (.37-.47) | .36 (.30-.41) | .23 (.18-.28) | .19 (.10-.31) | .21 (.11-.28) | .60 (.56-.64) |
|  |  |  |  |  |  |  |  |  |
| Time 2 | N=373 | N=354 | N=631 | N=555 | N=960 |  |  |  |
| Paranoia | .43 (.31-.54) | .16 (.02-.30) | .59 (.51-.66) | .36 (.26-.46) | .16 (.07-.24) | .52 (.42-.57) | .00 (.00-.07) | .48 (.43-.54) |
| Hallucinations | .40 (.28-.52) | .29 (.15-.42) | .61 (.53-.67) | .45 (.35-.54) | .21 (.13-.30) | .47 (.30-.58) | .06 (.00-.20) | .47 (.42-.53) |
| Cognitive disorganisation | .49 (.37-.59) | .27 (.13-.40) | .59 (.50-.65) | .26 (.14-.36) | .17 (.08-.26) | .53 (.44-.58) | .00 (.00-.06) | .47 (.42-.53) |
| Negative symptoms | .84 (.79-.87) | .40 (.27-.52) | .84 (.80-.87) | .64 (.57-.71) | .51 (.45-.58) | .62 (.53-.72) | .22 (.12-.31) | .16 (.14-.18) |
| Depression | .42 (.29-.53) | .32 (.19-.45) | .54 (.46-.61) | .33 (.22-.43) | .15 (.06-.23) | .50 (.38-.56) | .00 (.00-.08) | .50 (.45-.56) |

Note. MZM: monozygotic males; DZM: dyzygotic males; MZF: monozygotic females; DZF: dyzygotic females; DZOS: dyzygotic opposite sex; A: additive genetic influences; C: shared environmental influences; E: non-shared environmental influences. Anhedonia and grandiosity were not examined due to low correlations with depression.

**eTable3**. Information on the participating and non-participating families in the study.

|  | Participating | | Non-participating | |  |
| --- | --- | --- | --- | --- | --- |
| Male | 45% |  | 53% |  |  |
| Monozygotic | 35% |  | 32% |  |  |
| White | 94% |  | 91% |  |  |
| Mothers had one or more A-levels (UK advanced educational qualification) as highest qualification | 16% |  | 12% |  |  |
|  | M | SD | M | SD | *p* |
| SDQ Total scale, age 4 years | 8.54 | 4.44 | 9.41 | 4.70 | <.001 |
| SDQ Emotional problems subscale, age 4 years | 1.33 | 1.41 | 1.46 | 1.50 | <.001 |
| SDQ Total scale, age 12 years | 6.80 | 5.03 | 7.91 | 5.44 | <.001 |
| SDQ Emotional problems subscale, age 12 years | 1.81 | 1.91 | 1.93 | 2.03 | <.05 |

Note. SDQ, Strengths and Difficulties Questionnaire.

**eFigure 1:** Bivariate cross-lagged models for depression symptoms and psychotic experiences for males and females separately

Figure Note: Hall: hallucinations; cog dis: cognitive disorganisation. Additive genetic (A), shared (C) and non-shared environmental (E) influences on total variance at time 2 presented. Correlations between latent factors at time 2 presented on total variance.


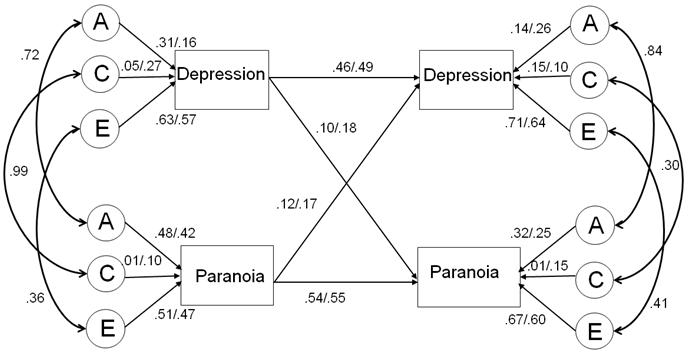


**eFigure 1a.** Longitudinal relationship between depression symptoms and paranoia (male/female)


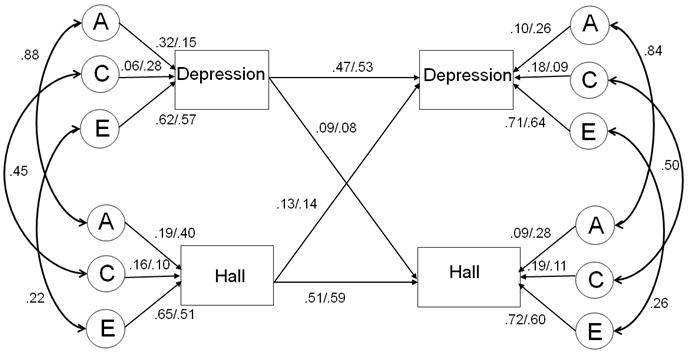


**eFigure 1b.** Longitudinal relationship between depression symptoms and hallucinations (male/female)


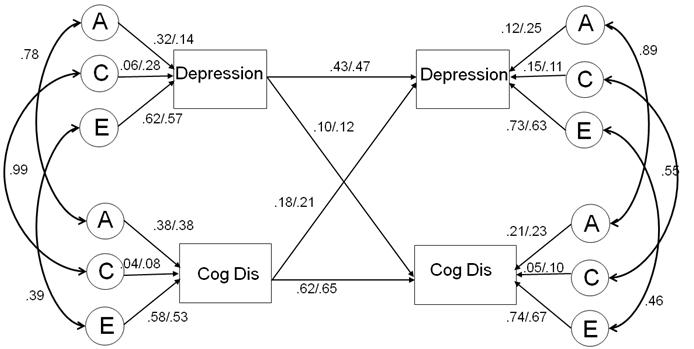


**eFigure 1c.** Longitudinal relationship between depression symptoms and cognitive disorganisation (male/female)
